# Skeletal muscle hypertrophy and enhanced mitochondrial bioenergetics following electrical stimulation exercises in spinal cord injury: a randomized clinical trial

**DOI:** 10.1007/s00421-024-05661-6

**Published:** 2024-11-22

**Authors:** Ashraf S. Gorgey, Refka E. Khalil, William Carter, Jeannie Rivers, Qun Chen, Edward J. Lesnefsky

**Affiliations:** 1 Spinal Cord Injury and Disorders, Spinal Cord Injury & Disorders Service, Richmond VA Medical Center, Richmond, VA USA; 2 Department of Physical Medicine & Rehabilitation, Virginia Commonwealth University, Richmond, VA USA; 3 General Surgery, Richmond VA Medical Center, Richmond, VA USA; 4 Department of Physiology and Biophysics, Richmond, VA USA; 5 Division of Cardiology, Department of Medicine, Pauley Heart Center, Richmond, VA USA; 6 Medical Service, Richmond VA Medical Center, Richmond, VA USA

**Keywords:** Neuromuscular electrical stimulation, Functional electrical stimulation, Resistance training, Electron transport chain, Mitochondrial complexes, Citrate synthase, Spinal cord injury

## Abstract

We examined the combined effects of neuromuscular electrical stimulation-resistance training (NMES-RT) and functional electrical stimulation-lower extremity cycling (FES-LEC) compared to passive movement training (PMT) and FES-LEC on mitochondrial electron transport chain (ETC) complexes and citrate synthase (CS) in adults with SCI**.** Thirty-two participants with chronic SCI were randomized to 24 weeks of NMES-RT + FES [n = 16 (14 males and 2 females) with an age range of 20–54 years old] or PMT + FES [n = 16 (12 males and 4 females) with an age range of 21–61 years old]. The NMES-RT + FES group underwent 12 weeks of surface NMES-RT using ankle weights followed by an additional 12 weeks of FES-LEC. The PMT + FES performed 12 weeks of passive leg extension movements followed by an additional 12 weeks of FES-LEC. Using repeated measures design, muscle biopsies of the vastus lateralis were performed at baseline (BL), post-intervention 1 (P1) and post-intervention 2 (P2). Spectrophotometer was used to measure ETC complexes (I-III) and CS using aliquots of the homogenized muscle tissue. Magnetic resonance imaging was used to measure skeletal muscle CSAs. A time effect was noted on CS *(P* = *0.001*) with an interaction between both groups *(P* = *0.01*). 46% of the participants per group had zero activities of CI without any changes following both interventions. A time effect was noted in CII (*P* = *0.023*) following both interventions. Finally, NMES-RT + FES increased CIII at P1 compared to BL (*P* = 0.023) without additional changes in P2 or following PMT + FES intervention. Skeletal muscle hypertrophy may potentially enhance mitochondrial bioenergetics after SCI. NMES-RT is likely to enhance the activities of complex III in sedentary persons with SCI. Clinical trials # NCT02660073.

## Background

Spinal cord injury (SCI) serves as a model of premature aging (Bauman & Spungen 1994; O'Brien et al. 2017c). Similarities between the two models have gained considerable interest in the research community. Persons with SCI experience metabolic inflexibility that is characterized by insulin resistance, glucose intolerance and the development of type II diabetes mellitus (Bauman & Spungen 2001, 2008). Following 40 years of age, a key characteristic feature is mitochondrial dysfunction (Ritov et al. 2010) that is likely to be associated with loss in muscle mass, altered body composition, increased ectopic adiposity and other metabolic derangements such as dyslipidemia and inflammatory cytokines after SCI (Spungen et al. 2003; O'Brien & Gorgey 2016; O'Brien et al. 2017c). Skeletal muscle mitochondrial electron transport chain (ETC) has previously been quantified after measuring citrate synthase (CS) enzyme [a biomarker of mitochondrial density] and complex III (CIII) activity in persons with SCI (Talmadge et al. 2002; O'Brien et al. 2017c, 2018). The authors noted strong relationships between CS or CIII activity and body composition parameters as well as biomarkers of metabolic profile in persons with SCI (O'Brien et al. 2017b, 2017c). Despite the lack of a control group, the findings demonstrated the significance of enhancing either mitochondrial density and/or activity to attenuate several of the cardio-metabolic risk factors in this population.

Exercise is a strong potent stimulus to stimulate mitochondrial biogenesis in the general population (Baldwin et al. 1972; Baar 2006; Carter et al. 2015). Even in paralyzed muscles, mitochondria demonstrated increased plasticity to metabolic demands in response to electrical stimulation exercise (Ryan et al. 2013; Erickson et al. 2017; Gorgey et al. 2020). Sixteen weeks of testosterone treatment (TT) with neuromuscular electrical stimulation-resistance training (NMES-RT) resulted in increasing CS and succinate dehydrogenase (SDH; a component of complex II) compared to TT only in persons with SCI (Gorgey et al. 2020). However, it is still unclear whether altering the paradigm of electrical stimulation exercise may influence mitochondrial biogenesis and predominately the enzymatic activities of the ETC in persons with SCI. Today, we have identified two major paradigms of electrical stimulation exercises that are commonly used in the rehabilitation of persons with SCI (Dolbow et al. 2023b). The first is NMES-RT and the second is functional electrical stimulation-lower extremity cycling (FES-LEC; FES) (Gorgey et al. 2023a, 2023b). The NMES-RT represents resistance exercise training, whereas FES is considered an alternative model of endurance training paradigm (Gorgey et al. 2023b).

We recently examined the hypothesis that combining these two paradigms of electrical stimulation exercises may result in greater attenuation of cardio-metabolic risk factors (Gorgey et al. 2023b). To test the hypothesis, we compared the effects of 12 weeks of passive movement training (PMT) and 12 weeks of FES training compared to 12 weeks of NMES-RT followed by 12 weeks of FES training, both groups underwent 24 weeks of supervised training (Gorgey et al. 2023b). Similar to previous findings, applications of NMES-RT + FES resulted in a greater VO_2_ peak following NMES-RT + FES compared to PMT + FES (Gorgey et al. 2021, 2023b). Robust muscle hypertrophy of the whole thigh muscle CSA, absolute thigh muscle CSA and knee extensor CSA were noted in the NMES-RT + FES group compared to PMT + FES. Furthermore, there is a decrease in visceral adipose tissue (VAT) following NMES-RT + FES. Other than evoking muscle hypertrophy, 12 weeks of FES training following either NMES-RT or PMT did not yield additional cardio-metabolic benefits in persons with SCI (Gorgey et al. 2023b).

Mechanistically, it is unclear whether altering the electrical stimulation exercise paradigms may induce different signaling pathways that impact different aspects of mitochondrial biogenesis. Both standard resistance and endurance training paradigms had been previously shown to positively enhance parameters of mitochondrial enzymatic electron transport chain (ETC) complexes (Ahmad et al. 2023). However, a portion of the disconnect between translating the findings between standard volitional exercises programs to electrical stimulation exercises is related primarily to differences in muscle recruitment behavior following electrical stimulation exercise as well as related fiber type distribution after SCI (Talmadge et al. 2002; Gregory & Bickel 2005). Resistance training is likely to enhance the hypertrophy signaling pathway [AMPK, Akt, mTOR]; whereas endurance training is likely to augment signaling pathway [AMPK, PGC1-alpha] that induces mitochondrial biogenesis (Porter et al. 2015; Gorgey et al. 2023a). Historically, the effects of RT have resulted in diluting mitochondrial content (Ploutz et al. 1994). However, RT demonstrated increases in maximal respiration without increasing mitochondrial mass (Porter et al. 2015; Groennebaek & Vissing 2017). After SCI, NMES-RT resulted in an increase in mitochondrial density and complex II activity without increasing mitochondrial respiration (Gorgey et al. 2020, 2021).

Therefore, the main goal of the current study was to determine whether evoking knee extensor (KE) muscle hypertrophy prior to FES-LEC training would further augment mitochondrial enzymatic activity than simply using FES-LEC only. We hypothesized that 12 weeks of NMES-RT + 12 weeks of FES would result in different mitochondrial enzymatic ETC adaptations compared to 12 weeks of PMT + 12 weeks of FES. Unlike our previous trial (Gorgey et al. 2020), we have provided detailed analysis of enzymatic activities of the ETC including complex I, II and III, NADH cytochrome *c* oxidoreductase (NCR) and NADH-ferricyanide oxidoreductase (NFR).

## Methods

### Study design

This is a follow-up trial to a recently published randomized controlled study that investigated the efficacy of NMES-RT + FES versus PMT + FES (control group) on cardio-metabolic risk factors in persons with chronic SCI (Gorgey et al. 2019, 2023b). After signing an approved consent form, participants were randomized into either 12 weeks of NMES-RT followed by 12 weeks of FES-LEC or PMT for 12 weeks followed by 12 weeks of FES-LEC. The entire duration of the study is 27 weeks (3 weeks of measurements and 24 weeks of training). Using repeated measures design, muscle biopsies were conducted at bassline (BL; prior starting any intervention), post-intervention 1 (P1; 12 weeks after intervention) and post-intervention 2 (P2; 24 weeks after intervention). The detailed inclusion–exclusion criteria were previously published (Gorgey et al. 2019, 2023b).

Thirty-two individuals, with chronic (≥ 1-year post injury) SCI were randomized into either NMES-RT + FES [n = 16; 14 males and 2 females] or PMT + FES [n = 16; 12 males and 4 females]. There were no differences in the physical and SCI characteristics of the participants between the NMES-RT + FES and PMT + FES groups as previously published and presented in Table 1 of that publication (Gorgey et al. 2023b).

**Table 1:** Citrate synthase (CS) and complexes I-III as well as CII + DUQ, NCR, NFR (mU/mg skeletal muscle protein) in persons with chronic SCI who were randomized into 24 weeks of either NMES-RT + FES or PMT-FES

	NMES-RT + FES-BL								PMT + FES-BL						
Baseline	CS	CI	CII	CII + DUQ	CIII	NCR	NFR	Baseline	CS	CI	CII	CII + DUQ	CIII	NCR	NFR
P003	95.0	3.6	2.4	14.0	88.1	13.6	No data	P001	86.0	9.1	No data	14.6	148.4	No data	No data
P004	110.0	1.6	4.1	19.8	185.1	11.1	384.0	P002	82.0	1.2	4.0	20.5	174.3	No data	No data
P005	51.0	2.1	1.1	9.3	129.1	4.5	292.0	P006	111.0	0.0	4.0	21.7	261.3	5.1	316.0
P007	107.0	0.0	3.7	21.8	201.9	14.2	278.0	P010	53.0	0.1	3.1	15.3	126.7	6.2	381.0
P012	56.0	0.0	2.8	8.3	59.7	8.2	241.0	P011	102.0	0.0	6.0	20.5	221.2	7.0	294.0
P014	91.0	0.0	4.7	21.0	182.8	3.7	302.0	P016	190.0	0.0	2.8	18.9	237.7	1.2	295.0
P015^#^	Low quality muscle biopsy tissues							P017	136.0	10.0	3.3	27.5	278.4	0.6	297.0
P019	127.0	3.0	2.4	14.2	255.0	2.0	255.0	P018	267.0	0.0	5.8	33.0	679.0	3.5	438.0
P020	54.0	0.0	0.7	10.8	118.7	0.1	40.0	P022	33.0	12.0	1.0	2.8	82.4	1.5	262.0
P024	52.0	0.0	1.3	11.6	194.0	6.3	170.0	P023	159.0	4.0	5.2	25.4	444.9	4.6	418.0
P028	303.0	0.0	2.7	27.0	377.8	1.0	937.0	P027	104.0	0.0	2.1	12.8	142.5	0.8	645.0
P029	79.0	4.0	1.9	1.7	122.7	6.1	396.0	P030	73.0	3.0	2.4	3.7	103.2	16.9	503.0
P032	48.0	7.0	0.8	0.0	142.0	8.4	157.0	P033	71.0	1.0	0.3	0.3	138.7	11.7	187.0
P035	92.0	7.0	3.1	1.2	162.7	3.7	519.0	P036	117.0	No data	4.4	No data	218.3	24.9	259.0
P037	110.0	0.0	3.0	8.7	156.4	9.2	345.0	P038	92.0	1.0	2.5	3.9	47.1	14.9	254.0
P040	70.0	1.0	1.1	3.4	83.0	2.0	424.0	P039	24.0	0.0	0.0	0.0	61.4	7.4	254.0
Mean	96.3	1.9	2.4	11.5	163.9	6.3	338.6	Mean	106.3	2.8	3.1	14.7	210.3	7.6	343.1
SD	65.1	2.5	1.3	8.4	86.0	4.6	227.8	SD	60.7	4.1	1.8	10.8	159.4	7.1	163.3

	NMES-RT + FES-P1								PMT + FES-P1						
Post-int. 1	CS	CI	CII	CII + DUQ	CIII	NCR	NFR	Post-int. 1	CS	CI	CII	CII + DUQ	CIII	NCR	NFR
P003	148.0	2.2	2.5	21.9	209.8	5.4	238.0	P001	89.0	0.0	3.6	19.4	186.7	5.2	334.0
P004	134.0	0.1	4.4	21.2	238.5	11.4	429.0	P002	72.0	0.0	3.6	16.4	125.3	8.3	220.0
P005	74.0	0.0	1.0	10.4	129.3	0.0	308.0	P006	148.0	0.0	5.2	27.0	249.7	9.2	355.0
P007	122.0	0.0	5.0	22.2	271.0	10.1	254.0	P010	63.0	0.0	3.4	16.8	146.4	10.0	363.0
P012	93.0	0.0	3.7	17.2	87.2	9.6	323.0	P011	90.0	0.2	3.7	19.6	237.8	9.4	325.0
P014	82.0	0.0	4.6	17.7	162.2	6.0	279.0	P016	216.0	3.0	4.5	19.5	527.1	2.6	295.0
P015	135.0	2.0	3.1	20.5	257.3	1.3	360.0	P017	109.0	2.0	2.1	11.3	301.6	1.0	226.0
P019	122.0	0.0	2.3	15.4	472.9	1.6	199.0	P018	177.0	1.0	3.3	21.9	481.1	1.3	278.0
P020	70.0	0.0	1.1	13.2	259.2	0.8	92.0	P022	34.0	9.0	0.3	2.2	29.0	5.9	114.0
P024	107.0	9.0	4.1	27.2	385.9	1.4	466.0	P023	134.0	0.0	4.8	24.7	473.0	2.9	312.0
P028	347.0	0.0	1.2	25.7	397.3	0.0	621.0	P027	107.0	0.0	1.7	11.7	159.6	0.0	691.0
P029	97.0	5.0	2.9	3.1	142.1	5.0	491.0	P030	75.0	0.0	2.6	2.2	230.6	35.3	305.0
P032	95.0	2.0	2.1	1.0	257.2	9.8	240.0	P033	87.0	0.0	1.8	1.7	222.4	17.1	401.0
P035	102.0	3.0	3.4	2.0	208.4	0.0	492.0	P036	119.0	0.0	3.9	11.7	184.1	3.8	325.0
P037	112.0	0.0	3.9	13.5	132.0	14.2	329.0	P038	72.0	0.0	2.1	2.3	74.9	9.2	205.0
P040	Early withdrawal because of COVID-19 Pandemic							P039	31.0	1.0	0.2	0.6	78.1	5.7	313.0
Mean	122.7	1.5	3.0	15.5	240.7	5.1	341.4	Mean	101.4	1.0	2.9	13.1	231.7	7.9	316.4
SD	66.1	2.6	1.3	8.3	109.2	4.8	137.2	SD	49.2	2.3	1.5	9.0	148.4	8.5	122.3

	NMES-RT + FES-P2								PMT + FES-P2						
Post-int. 2	CS	CI	CII	CII + DUQ	CIII	NCR	NFR	Post-int. 2	CS	CI	CII	CII + DUQ	CIII	NCR	NFR
P003	169.0	0.0	6.1	27.6	294.7	13.4	288.0	P001	105.0	0.0	4.4	26.3	223.4	8.8	373.0
P004	119.0	1.1	4.5	22.2	260.1	16.1	403.0	P002	143.0	0.0	6.8	32.0	337.5	9.0	341.0
P005	62.0	0.3	2.4	9.9	118.7	4.7	344.0	P006	Withdrawal after P1						
P007	100.0	0.7	4.4	19.4	202.7	9.3	216.0	P010	131.0	0.2	6.0	29.5	247.8	8.3	471.0
P012	89.0	0.1	4.7	21.8	161.4	4.1	313.0	P011	136.0	0.8	5.6	29.4	282.8	8.8	405.0
P014	93.0	1.2	2.3	14.4	114.0	2.3	411.0	P016	244.0	2.0	5.1	32.2	671.5	0.6	436.0
P015	135.0	7.0	3.6	20.5	406.3	1.5	325.0	P017	189.0	5.0	4.9	31.0	554.1	3.4	510.0
P019	160.0	2.0	4.2	24.4	501.5	3.1	245.0	P018	203.0	0.0	5.7	27.8	630.3	2.9	345.0
P020	61.0	1.0	0.8	3.1	209.7	0.6	34.0	P022	51.0	8.0	0.0	0.7	10.6	4.5	160.0
P024	84.0	4.0	1.1	10.4	296.5	0.0	188.0	P023	Withdrawal after P1						
P028	Withdrawal after P1							P027	120.0	0.0	2.5	14.3	148.1	0.0	465.0
P029	81.0	0.0	2.6	2.4	148.2	8.7	456.0	P030	82.0	0.0	2.2	1.6	79.4	1.0	291.0
P032	93.0	0.0	2.6	0.3	189.1	8.3	228.0	P033	145.0	0.0	3.2	3.8	254.3	8.7	435.0
P035	Withdrawal after P1							P036	94.0	4.0	3.2	5.1	156.9	13.9	255.0
P037	113.0	0.0	3.5	11.0	152.6	14.7	379.0	P038	74.0	0.0	1.8	2.1	80.3	12.3	172.0
P040	Early withdrawal because of COVID-19 Pandemic							P039	36.0	0.0	0.0	1.4	73.3	8.0	303.0
Mean	104.5	1.2	3.3	14.4	235.0	6.7	294.6	Mean	125.2	1.3	3.7	16.9	267.9	6.5	354.4
SD	33.7	2.1	1.5	9.0	115.5	5.5	113.5	SD	65.0	2.5	2.3	14.0	215.8	4.5	139.2

*Complex I* NADH:ubiquinone oxidoreductase

*Complex II* Succinate:ubiquinone oxidoreductase

*Complex II* + *DUQ* Complex II with exogenous decylubiquinone

*Complex III* Ubiquinol-cytochrome c oxidoreductase

*NCR* NADH cytochrome c oxidoreductase

*NFR* NADH-ferricyanide oxido-reductase

#, 015-BL had low quality muscle tissue to be homogenized for ETC analysis

### Exercise interventions

#### a. NMES-resistance training (NMES-RT)

The NMES-RT + FES group underwent 12 weeks of NMES-RT followed by 12 weeks of FES-LEC in a lab-based setting under full supervision. The NMES-RT was conducted as previously described to induce concentric-eccentric actions. Two carbon (8 × 10 cm^2^) adhesive gel electrodes were placed over the skin of the knee extensor muscle group. Electrodes were replaced every 4 weeks to ensure proper adherence and appropriate electrical conductivity. Stimulation parameters were set at a frequency of 30 Hz, biphasic pulses of 450 µs with interpulse interval of 50 µs and amplitude of current sufficient to evoke knee extension. The amplitudes of the current (mA) for every single repetition were rerecorded for the entire session. Training was performed twice weekly of 4 sets × 10 repetitions, separated by at least 48 h, for 12 weeks while participants seated in their wheelchairs. Training sets (4 sets per leg) were alternated between the right and left knee extensors and separated by 2 min of rest progression of the training started with the first week without ankle weights to ensure leg extension against gravity and progressed in an increments of 2 lbs. per leg on weekly basis once 40 repetitions of leg extension were attained (Ryan et al. 2013; Gorgey et al. 2023a, 2023b).

#### b. Passive movement training (PMT)

The PMT + FES underwent 12 weeks of passive movement training (PMT) followed by 12 weeks of FES-LEC (Gorgey et al. 2019, 2021). PMT training was matched with NMES-RT on sets (4 sets) and repetitions (10 reps per set) of the training. The design ensured testing the primary hypothesis that evoking skeletal muscle hypertrophy via NMES-RT may enhance mitochondrial density (CS) and complexes (I–III) compared to PMT. A member of the research team supported the leg proximal to the ankle joints and moved it from 90$^\circ$ knee flexion close to full knee extension. The leg was maintained up for 5 s and returned down for 5 s. The passive movements were repeated in the same fashion described in NMES-RT protocol: 10 reps for the right leg followed by 10 reps for the left leg for total of 4 sets × 10 reps.

#### c. Functional electrical stimulation-lower extremity cycling (FES-LEC)

Participants in both groups (NMES-RT-FES or PMT + FES) underwent additional 12 weeks of FES-LEC training (Gorgey et al. 2019, 2021; Bekhet et al. 2022). This allowed a test of the hypothesis that preceding NMES-RT may condition the muscles during FES-LEC to attenuate muscle fatigue and enhance mitochondrial capacity as a primary mode of endurance training. FES-LEC was conducted for 12 weeks, twice weekly, for each participant. Rectangular adhesive conductive electrodes were placed on the skin of the knee extensor, hamstrings, and gluteus maximus muscle groups. Pulse frequency was set at 33.3 Hz, pulse duration at 350 µs and resistance was adjusted every 10 min to maintain a speed of 40–45 revolutions per minute (RPM). The resistance of the bike was increased in 0.5 Nm increments per 10-min stage over the course of 12 weeks. The progression in resistance was customized based on the subject’s performance riding the FES-LEC ergometer over 12 weeks (Gorgey et al. 2019). The fatigue threshold was set at 18 RPM; if RPM falls below 18 RPM; the bike was set to automatically shift from active to passive cycling (cool-down). During the 3-min cool-down period, participants passively cycled with no electrical stimulation. The cool down period was then followed by 5 min of recovery, during which the participant was still connected to the bike but in a complete resting position while constantly monitoring blood pressure and heart rate.

### Measurements

#### a. Body weight and body mass index (BMI) and Anthropometrics

Each participant was asked to empty their bladder and then propel onto a wheelchair weighing scale to evaluate weight in kg. The wheelchair was measured separately, and the difference taken for the final body weight. The height of each participant was determined with the subject on his/her right side in the supine position. Two smooth wooden boards were placed at the participant’s head and heels and the distance between them determined the height in nearest cm. The BMI (kg/m^2^) was calculated as weight (kg)/height^2^ (m^2^) (Gater et al. 2021).

#### b. Magnetic resonance imaging (MRI)

Skeletal muscle cross-sectional area (CSAs were determined before (baseline), and twice after 12 week interventions (post-intervention 1 and post-intervention 2) using a 1.5 Tesla GE magnet (Gorgey et al. 2021, 2023a). Transaxial images, 0.8 cm thick and 1.6 cm apart, were taken from the hip joint to the knee joint (thigh). Image J-software was carefully used to match the images at different time points [BL, P1 and P2]. WinVeseel 2.011 was used to analyze whole thigh muscle CSA and absolute muscle CSA to determine the changes overtime in persons with SCI (Gorgey et al. 2021, 2023a).

#### c. Mitochondrial enzyme assays

Biopsy samples of the right vastus lateralis muscle were obtained by a 14-gauge Tru‐cut™ biopsy needle, immediately frozen in liquid nitrogen, and stored at − 70 °C until analysis. Muscle biopsies were captured 5–7 days after the last training bouts in both groups for P1 and P2 measurements. A portion of this sample (~ 10–25 mg) was homogenized in 220 mmol/L mannitol, 70 mmol/L sucrose, 5 mmol/L MOPS, 2 mmol/L EDTA, with complete™ protease inhibitor cocktail (Sigma‐Aldrich), pH 7.4. Samples were centrifuged at 2000 rpm (371* g*) for 5 min at 4 °C and the supernatant was used for analysis. After protein concentration was determined by the Lowry method, samples were solubilized in 1% potassium cholate (Lesnefsky et al. 1997; Brass et al. 2001). The samples were analyzed on the same day as homogenization.

Mitochondrial enzyme activity was assessed spectrophotometrically as previously described by our group and others (Lesnefsky et al. 1997; O'Brien et al. 2017b, a; O'Brien et al. 2018). Enzyme activity was measured spectrophotometrically at 37 °C using a Hewlett‐Packard diode array spectrophotometer. Individual enzymes of ETC complexes I-III were assessed. Complex I (CI; NADH:ubiquinone oxidoreductase) was measured as the rotenone-sensitive oxidation of NADH with decylubiquinone as an electron acceptor. Rotenone-sensitive NADH cytochrome *c* reductase (NCR; NADH → endogenous ubiquinone/ubiquinol → complex III → exogenous cytochrome *c*) was also measured to reflect complex I and III activities. Complex II (CII, Succinate:ubiquinone oxidoreductase) was measured as the TTFA (thenoyltrifluoroacetone)-sensitive reduction of the DCPIP (2,6 dichlorophenol-indophenol) in the absence or presence of decylubiquinone (DUQ). Complex III (CIII; Ubiquinol-cytochrome *c* oxidoreductase) activity was determined as the antimycin A‐sensitive increase in absorbance at 550 nm for 45 s, representing the reduction in cytochrome *c* coupled to the oxidation of ubiquinol to ubiquinone as previously described (Brass et al. 2001). We also measured the enzymatic activity of NADH-ferricyanide-reductase (NFR) as an index of the activity of the initial segment of complex I, the NADH dehydrogenase portion of the complex. Ferricyanide accepts electrons from the FMN cofactor of complex I, located in the initial portion of electron flow through complex I.

Citrate synthase (CS) activity was measured as an index of mitochondrial mass. Citrate synthase (CS) was measured by the formation of the thionitrobenzoate anion with absorbance measured at a wavelength of 412 nm for 90 s after addition of 5,5‐dithiobis‐(2,4‐nitrobenzoic acid), acetyl‐CoA, and oxaloacetate as previously described (Brass et al. 2001). Absorbance was measured before and after the addition of oxaloacetate and background absorbance was subtracted from the final reading. Each sample was run in duplicate or triplicate for each assay, depending on the amount of sample available. Data were converted from arbitrary units per minute to nmol/min by using the extinction coefficients of 13.6 mM^−1^ cm^−1^ for thionitrobenzoate, and 19.1 mM^−1^ cm^−1^ for cytochrome *c*, and 6.23 mM^−1^ cm^−1^ for NADH. Data were presented as absolute values and following normalization by CS.

#### d. Dietary recalls

Each participant met with a dietitian at the start of the study and was asked to maintain a weekly 3- to 5-day food dietary log to monitor their caloric and liquid intake for the duration of the study (Gorgey et al. 2015). Dietary logs were administered to ensure controlling for the caloric intake and macronutrients. No nutritional advice was given on portion size of the food. However, based on participants’ basal metabolic rate, the dietitian recommended the percentage of macronutrients at 45% carbohydrates, 30% fat and 25% total protein. Dietary logs were analyzed on a weekly basis using a nutritional software package (Nutrition Data System for Research version 2014) under the supervision of a registered dietitian. After the analysis was completed, the average caloric intake (kcal) and percentage macronutrients (carbohydrates, fats and proteins) were calculated and monthly feedback was provided via phone call.

### Statistical analyses

All data were tested for normality using the Shapiro–Wilk tests. Outliers were detected using normal Q-Q plots at different time points (BL, P1, P2) for each group. If normality was not assumed (*P* < 0.05), the examined variable was then log-transformed before conducting any statistical analyses. Independent T-tests were conducted to examine the differences in BL between NMES-RT + FES and PMT + FES groups. Multivariate analysis of covariance (MANCOVA) was conducted to statistically examine the effects of NMES-RT + FES vs. PMT + FES intervention on mitochondrial ETC after using the baseline measurements as a covariate. If ANCOVA assumptions were not assumed, mixed model analysis of variance (MANOVA) was then used to determine whether there a time effect (BL, P1 and P2), between group effects or interaction. Statistical analyses were performed using IBM-SPSS version 29.0 (SPSS, Chicago, IL). Statistical significance was set at alpha level of 0.05 and all values were presented as mean ± SD.

## Results

Overall, participants enrolled and participated in the current trial were 32 participants. Physical characteristics [age: 39 ± 12 vs. 42 ± 14 yrs.; *P* = 0.5, weight: 71 ± 18 vs. 68 ± 13 kg;* P* = 0.7 and BMI: 23 ± 6.0 vs. 23 ± 4.0 kg/m^2^; *P* = 0.68] were not different between NMES-RT + FES and PMT + FES, respectively.

Muscle biopsy data are presented for 31 participants at BL, P 1 [NMES-RT + FES; n = 15 and PMT + FES: n = 16] and 27 participants at P 2 [NMES-RT + FES; n = 13 and PMT + FES: n = 14]. The factors of withdrawals from the trial are listed in Table 1.

Statistical analyses using MANCOVA were completed on 26 participants at BL, P1 and P2 [NMES-RT + FES; n = 12 and PMT + FES: n = 14]. In all participants, muscle biopsies were performed of the right VL muscle in both groups; except in 1 participant the left VL muscle was performed in the NMES-RT + FES group. There were no between group differences in any of the examined interventions on parameters of mitochondrial bioenergetics. Individual data for all the studied variables are presented in Table 1. Similarly, adjustment of all enzymatic complex activities to CS results in non-significant effects following both NMES-RT or FES-LEC (Table 2). Data from the dietary recalls were previously published and ensured equivalent caloric and macronutrient intakes between both groups (Gorgey et al. 2023b).

**Table 2:** Complexes I-III as well as CII + DUQ, NCR, NFR (mU/mg skeletal muscle protein/CS) after adjustment to CS in persons with chronic SCI who were randomized into 24 weeks of either NMES-RT + FES or PMT-FES

	NMES-RT + FES-BL						PMT + FES-BL						
Baseline	CI	CII	CII + DUQ	CIII	NCR	NFR	Baseline	CI	CII	CII + DUQ	CIII	NCR	NFR
P003	0.04	0.025	0.147	0.93	0.14	No data	P001	0.10	No data	0.17	1.73	No data	No data
P004	0.01	0.037	0.180	1.68	0.10	3.49	P002	0.01	0.05	0.25	2.13	No data	No data
P005	0.04	0.021	0.183	2.53	0.09	5.73	P006	0.00	0.04	0.20	2.35	0.05	2.85
P007	0.00	0.034	0.203	1.89	0.13	2.60	P010	0.00	0.06	0.29	2.39	0.12	7.19
P012	0.00	0.050	0.148	1.07	0.15	4.30	P011	0.00	0.06	0.20	2.17	0.07	2.88
P014	0.00	0.052	0.231	2.01	0.04	3.32	P016	0.00	0.01	0.10	1.25	0.01	1.55
P015^#^	Low quality muscle biopsy samples						P017	0.07	0.02	0.20	2.05	0.00	2.18
P019	0.02	0.019	0.112	2.01	0.02	2.01	P018	0.00	0.02	0.12	2.54	0.01	1.64
P020	0.00	0.012	0.199	2.20	0.00	0.74	P022	0.36	0.03	0.08	2.50	0.05	7.94
P024	0.00	0.026	0.223	3.73	0.12	3.27	P023	0.03	0.03	0.16	2.80	0.03	2.63
P028	0.00	0.009	0.089	1.25	0.00	3.09	P027	0.00	0.02	0.12	1.37	0.01	6.20
P029	0.05	0.024	0.022	1.55	0.08	5.01	P030	0.04	0.03	0.05	1.41	0.23	6.89
P032	0.15	0.02	0.00	2.96	0.17	3.27	P033	0.01	0.00	0.00	1.95	0.16	2.63
P035	0.08	0.034	0.013	1.77	0.04	5.64	P036	No data	0.04	No data	1.87	0.21	2.21
P037	0.00	0.027	0.079	1.42	0.08	3.14	P038	0.01	0.03	0.04	0.51	0.16	2.76
P040	0.01	0.016	0.048	1.19	0.03	6.06	P039	0.00	0.00	0.00	2.56	0.31	10.58
Mean	0.03	0.03	0.13	1.88	0.08	3.69	Mean	0.04	0.03	0.13	1.97	0.10	4.30
SD	0.04	0.01	0.08	0.87	0.06	1.89	SD	0.09	0.02	0.09	0.60	0.10	3.05

	NMES-RT + FES-P1						PMT + FES-P1						
Post-int. 1	CI	CII	CII + DUQ	CIII	NCR	NFR	Post-int. 1	CI	CII	CII + DUQ	CIII	NCR	NFR
P003	0.015	0.017	0.148	1.418	0.036	1.608	P001	0.00	0.04	0.22	2.10	0.06	3.75
P004	0.000	0.033	0.159	1.780	0.085	3.201	P002	0.00	0.05	0.23	1.74	0.12	3.06
P005	0.000	0.013	0.140	1.747	0.000	4.162	P006	0.00	0.04	0.18	1.69	0.06	2.40
P007	0.000	0.041	0.182	2.221	0.083	2.082	P010	0.00	0.05	0.27	2.32	0.16	5.76
P012	Outliers						P011	0.00	0.04	0.22	2.64	0.10	3.61
P014	0.000	0.056	0.215	1.978	0.073	3.402	P016	0.01	0.02	0.09	2.44	0.01	1.37
P015	0.015	0.023	0.152	1.906	0.010	2.667	P017	0.02	0.02	0.10	2.77	0.01	2.07
P019	0.000	0.019	0.126	3.876	0.013	1.631	P018	0.01	0.02	0.12	2.72	0.01	1.57
P020	0.000	0.016	0.189	3.703	0.011	1.314	P022	0.26	0.01	0.06	0.85	0.17	3.35
P024	0.084	0.039	0.254	3.607	0.013	4.355	P023	0.00	0.04	0.18	3.53	0.02	2.33
P028	0.000	0.004	0.074	1.145	0.000	1.790	P027	0.00	0.02	0.11	1.49	0.00	6.46
P029	0.052	0.030	0.032	1.465	0.052	5.062	P030	0.00	0.03	0.03	3.07	0.47	4.07
P032	0.021	0.022	0.011	2.707	0.103	2.526	P033	0.00	0.02	0.02	2.56	0.20	4.61
P035	0.029	0.034	0.019	2.043	0.000	4.824	P036	0.00	0.03	0.10	1.55	0.03	2.73
P037	0.000	0.035	0.120	1.179	0.127	2.938	P038	0.00	0.03	0.03	1.04	0.13	2.85
P040	Early withdrawal because of the COVID-19 Pandemic						P039	0.03	0.01	0.02	2.52	0.18	10.10
Mean	0.02	0.03	0.13	2.20	0.04	2.97	Mean	0.02	0.03	0.12	2.19	0.11	3.75
SD	0.03	0.01	0.07	0.93	0.04	1.25	SD	0.07	0.01	0.08	0.74	0.12	2.19

	NMES-RT + FES-P2							PMT + FES-P2					
Post-int. 2	CI	CII	CII + DUQ	CIII	NCR	NFR	Post-int. 2	CI	CII	CII + DUQ	CIII	NCR	NFR
P003	0.00	0.04	0.16	1.74	0.08	1.70	P001	0.00	0.04	0.25	2.13	0.08	3.55
P004	0.01	0.04	0.19	2.19	0.14	3.39	P002	0.00	0.05	0.22	2.36	0.06	2.38
P005	0.01	0.04	0.16	1.91	0.08	5.55	P006	Withdrawal after P1					
P007	0.01	0.04	0.19	2.03	0.09	2.16	P010	0.00	0.05	0.23	1.89	0.06	3.60
P012	Outliers						P011	0.01	0.04	0.22	2.08	0.06	2.98
P014	0.01	0.02	0.15	1.23	0.02	4.42	P016	0.01	0.02	0.13	2.75	0.00	1.79
P015	0.05	0.03	0.15	3.01	0.01	2.41	P017	0.03	0.03	0.16	2.93	0.02	2.70
P019	0.01	0.03	0.15	3.13	0.02	1.53	P018	0.00	0.03	0.14	3.10	0.01	1.70
P020	0.02	0.01	0.05	3.44	0.01	0.56	P022	0.16	0.00	0.01	0.21	0.09	3.14
P024	0.05	0.01	0.12	3.53	0.00	2.24	P023	Withdrawal after P1					
P028	Withdrawal after P1						P027	0.00	0.02	0.12	1.23	0.00	3.88
P029	0.00	0.03	0.03	1.83	0.11	5.63	P030	0.00	0.03	0.02	0.97	0.01	3.55
P032	0.00	0.03	0.00	2.03	0.09	2.45	P033	0.00	0.02	0.03	1.75	0.06	3.00
P035	Withdrawal after P1						P036	0.04	0.03	0.05	1.67	0.15	2.71
P037	0.00	0.03	0.10	1.35	0.13	3.35	P038	0.00	0.02	0.03	1.09	0.17	2.32
P040	Early withdrawal because of the COVID-19 Pandemic						P039	0.00	0.00	0.04	2.04	0.22	8.42
Mean	0.01	0.03	0.12	2.29	0.06	2.95	Mean	0.02	0.03	0.12	1.87	0.07	3.26
SD	0.02	0.01	0.06	0.79	0.05	1.58	SD	0.04	0.02	0.09	0.92	0.07	1.78

*Complex I* NADH:ubiquinone oxidoreductase

*Complex II* Succinate:ubiquinone oxidoreductase

*Complex II* + *DUQ* Complex II with exogenous decylubiquinone

*Complex III* Ubiquinol:cytochrome *c* oxidoreductase

*NCR* NADH:cytochrome *c* oxidoreductase

*NFR* NADH:ferricyanide oxidoreductase

#, 015-BL had low quality muscle tissue to be homogenized for ETC analysis

### Effects of NMES-RT + FES vs. PMT + FES on muscle CSA

**Fig. 1:**
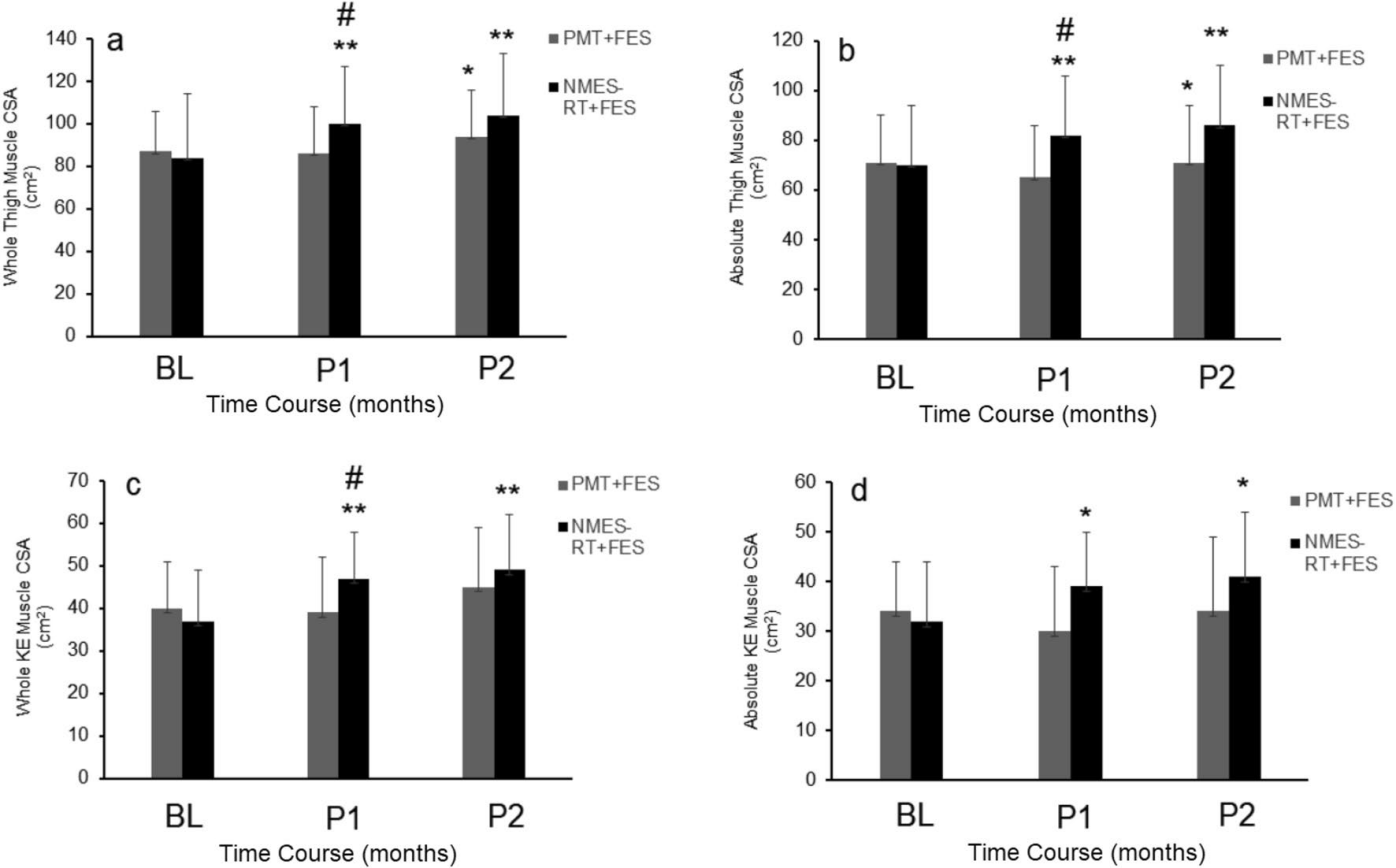
MRI changes of whole and absolute right thigh and knee extensor muscle CSA in persons with complete SCI at baseline (BL), P1 and P2. **, time effect, *P* < 0.001; *, time effect, P < 0.05; #, between group effect *P* < 0.05

The entire results of the changes in muscle size were previously published in detail (Gorgey et al. 2023b). Figure 1a, b demonstrates the changes of the right whole and absolute muscle CSA of the whole thigh and the right knee extensor muscle group. NMES-RT + FES resulted in increasing whole thigh muscle CSA by 19% (*P* < 0.001) and 24% (*P* < 0.001) as well as absolute thigh muscle CSA by 17% and 23% (*P* < 0.05) at P1 and P2, respectively. PMT + FES increased only right whole thigh muscle CSA by 8% (*P* < 0.05) at P2. There was between group differences in whole thigh muscle CSA (*P* < 0.001) and absolute muscle CSA (*P* < 0.05) at P1 between NMES-RT + FES and PMT + FES.

For KE extensor muscle group, NMES-RT + FES resulted in increasing whole KE muscle CSA by 27% (*P* < 0.001) and 32% (*P* < 0.001) as well as absolute KE muscle CSA by 22% (*P* < 0.05) and 28% (*P* < 0.05) at P1 and P2, respectively (Fig. 1c &d). PMT + FES increased the right whole KE muscle CSA by 12.5% (*P* < 0.05) at P2. There was a between group difference in the whole KE muscle CSA (*P* < 0.001) at P1 between NMES-RT + FES and PMT + FES.

### Effects of NMES-RT + FES vs. PMT + FES on citrate synthase

**Fig. 2:**
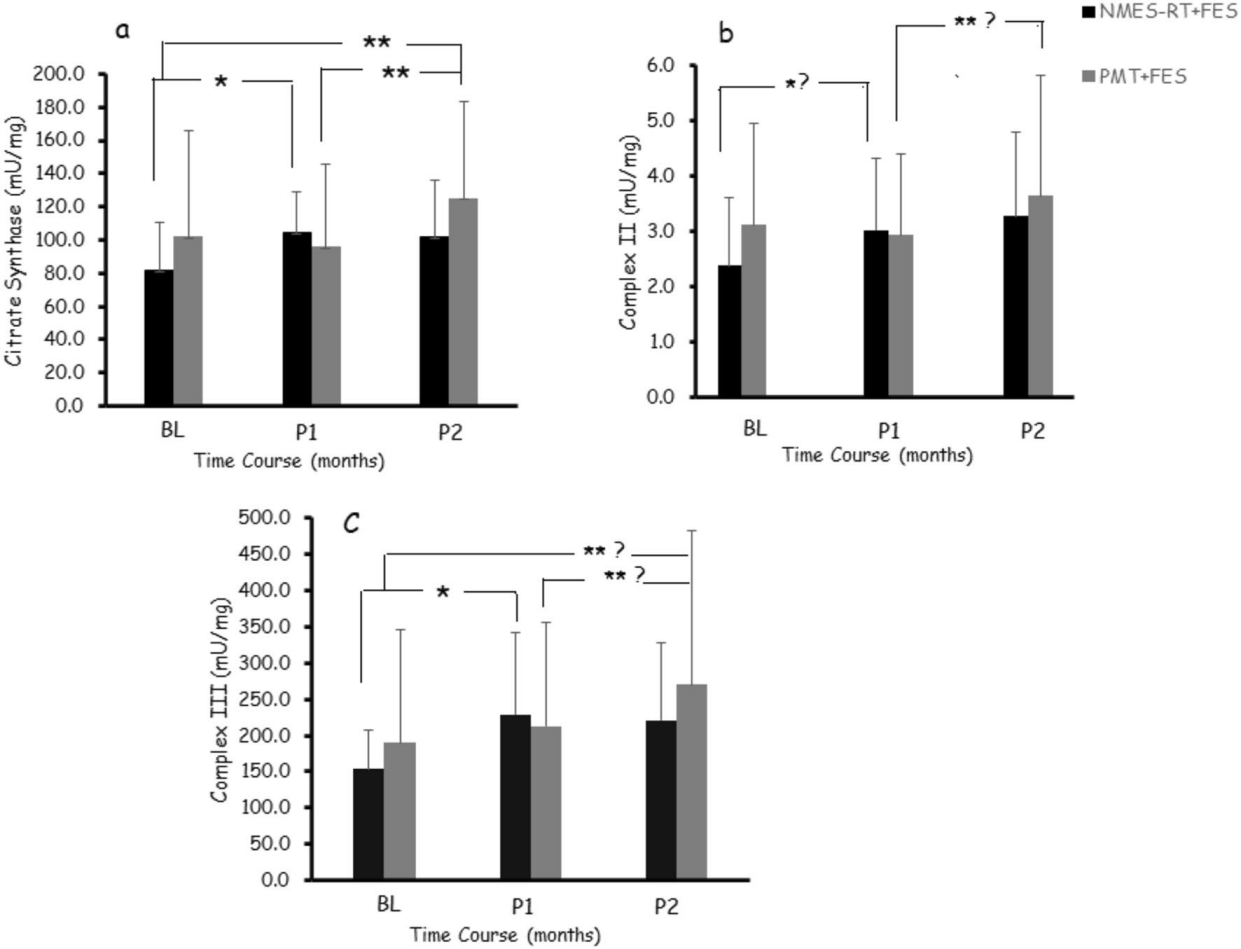
Time course of changes in a) CS, b) CII and c) CIII following 24 weeks of NMES-RT + FES or PMT + FES in persons with chronic SCI. Measurements were captured at BL (Prior to intervention), P1 (12 weeks post NMES-RT or PMT) and P2 (12 weeks post FES-LEC in both groups), *, statistically significant difference in NMES-RT + FES following P1; **, statistically significant differences in PMT + FES in P2 compared to BL or P1; **?, trend of statistical difference following PMT + FES in P2 compared to BL or P1

Both interventions demonstrated a time effect on CS *(P* = *0.001; η*^*2*^_p_ = 0.24) with interaction between both groups *(P* = *0.01; η*^*2*^_p_ = 0.17; Fig. 2a). Pairwise comparisons demonstrated that NMES-RT + FES increased CS at P1 compared to BL (*P* = 0.012; 28%) without additional changes in P2 compared to P1 (*P* = 1.0) or BL (*P* = 0.14). The PMT + FES demonstrated no changes in P1 (*P* = 1) and showed increases in CS in P2 compared to P1 (*P* = 0.049; 24%) and BL (*P* = 0.001; 18%).

**Fig. 3:**
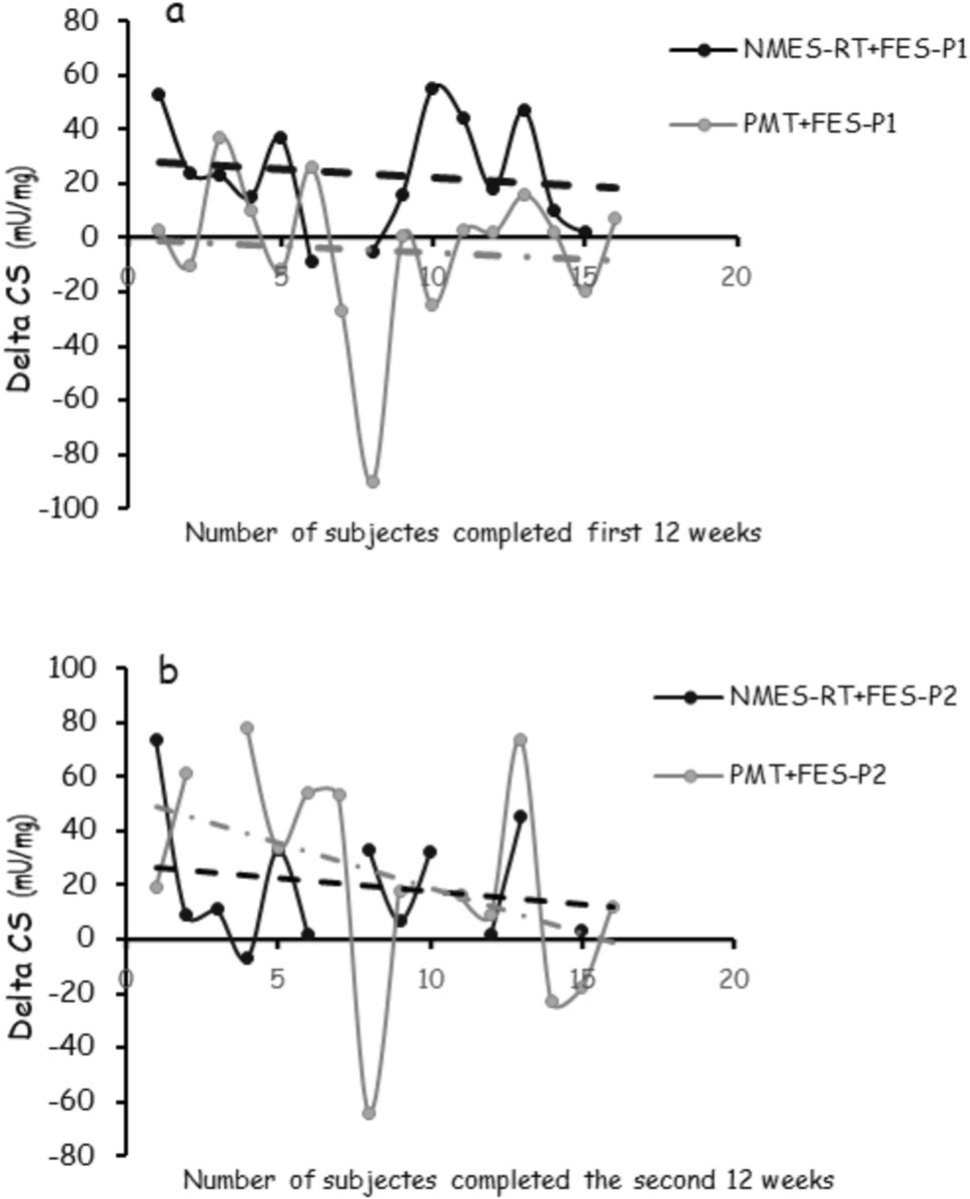
Individual data points in citrate synthase (CS) between NMES-RT + FES or PMT + FES in persons with chronic SCI. **a)** Following P1, there is 23.5 units increase in CS in the NMES-RT + FES compared to decrease in the PMT + FES; **b)** In P2, the addition of 12 weeks of FES-LEC reciprocated CS in the PMT + FES group without additional changes in the NMES-RT + FES group. Dashed black (**––**) and grey (––) lines represent the trend of changes in CS following NMES-RT + FES and PMT + FES, respectively, in P1 and P2

Because of the interaction in CS, mean differences revealed 23.5 ± 21.0 vs.−5.0 ± 2.5 mu/mg in the NMES-RT + FES vs. PMT + FES in P1 (*P* = 0.004; Fig. 3a). Following P2, mean differences in CS reciprocated in the PMT + FES [23.5 ± 40.0 mu/mg] without additional changes in the NMES-RT + FES [20.0 ± 23.0 mu/mg] (Fig. 3b); however, it did not attain statistical differences (*P* = 0.8).

### Effects of NMES-RT + FES vs. PMT + FES on complexes I-III

Analyses of complex I revealed low quality data at BL [NMES-RT + FES; n = 15 and PMT + FES: n = 15], P1[NMES-RT + FES; n = 15 and PMT + FES: n = 16] and P2 [NMES-RT + FES; n = 13 and PMT + FES: n = 14]. As highlighted in Table 1, seven out of the 15 (46%) participants per group had zero activities of CI in the NMES-RT + FES and PMT + FES at BL. This may suggest diminished activities of CI in the muscles of persons with SCI. The lower activities of CI did not change following either interventions at P1 or P2. Independent t-tests at each time points revealed no statistical changes between both groups [BL: *P* = *0.6*; P1: *P* = *0.79*; P2: *P* = *0.33*]. Both interventions did not influence NFR activities, a subunit of complex I, at any time points during the study.

A time effect was noted in Complex II (*P* = *0.023; η*^*2*^_p_ = 0.15) following both NMES-RT + FES and PMT + FES (Fig. 2b). Pairwise comparisons revealed a trend towards increasing CII activity at P1 compared to BL (*P* = 0.072; 31%) and P2 compared to P1 (*P* = 0.085; 40%) following NMES-RT + FES and PMT + FES, respectively. Furthermore, CII plus exogenous Q (CII + DUQ) combined showed time effect following both interventions (*P* = *0.032; η*^*2*^_p_ = 0.14) with a strong interaction effect (*P* = *0.009; η*^*2*^_p_ = 0.19). Pairwise comparisons suggested a trend of increasing CII + DUQ (*P* = 0.09) at P1 compared to BL in the NMES-RT + FES group. The PMT + FES demonstrated robust increases in CII + DUQ in P2 compared to P1(*P* = 0.006) and a trend in P2 compared to BL (*P* = 0.08).

The complex III data were log-transformed prior to statistical analysis (Fig. 2c). A clear time effect was noted in CIII *(P* = *0.027; η*^*2*^_p_ = 0.14) following both NMES-RT + FES and PMT + FES interventions without interaction effect *(P* = *0.45)*. Pairwise comparisons demonstrated that NMES-RT + FES increased CIII at P1 compared to BL (*P* = 0.023; 50%) without additional changes in P2 compared to P1 (*P* = 1.0; − 3.5%) or BL (*P* = 0.23; 44%). The PMT + FES demonstrated no changes in CIII activities at either P1 or P2. Finally, NCR and NFR did not change following either NMES-RT + FES or PMT + FES in persons with SCI.

## Discussion

The major finding of the current work is that skeletal muscle hypertrophy either via NMES-RT + FES or PMT + FES is a key determinant of increasing mitochondrial bioenergetics as measured by CS, a marker of mitochondrial density, and complexes (CII, CII + DUQ and CIII) in persons with SCI. Persons with SCI suffer from complex I deficiency which is the primary entry of the ETC and likely to contribute skeletal muscle redox imbalance (see below). Similar to recent published findings, the addition of 12 weeks of FES-LEC to 12 weeks of NMES-RT did not further augment mitochondrial bioenergetics in persons with SCI. NMES-RT + FES resulted in further enhancement in complex III activity but not following PMT + FES training.

### Significance and rationale of the work

Recently, we investigated the effects of NMES-RT + FES compared to PMT + FES on cardiometabolic risk factors (Gorgey et al. 2023b). We noted that the addition of 12 weeks of FES-LEC following 12 weeks of NMES-RT did not result in an additional increase in muscle size. There was an increase in muscle mass after adding 12 weeks of FES-LEC to PMT. The addition of FES-LEC resulted in recognized gains in power and resistance only in P2 in the NMES-RT + FES. NMES-RT + FES managed to increase leg VO_2_ peak compared to PMT; however, the addition of FES-LEC resulted in increasing VO_2_ and relative VO_2_ in P2 compared to P1 in both groups. Furthermore, NMES-RT + FES resulted in a decrease in the LDL-C level as well as total trunk VAT CSA (Gorgey et al. 2023b).

Recent guidelines have recommended both aerobic and resistance training to evoke muscle hypertrophy, strength or increasing aerobic capacity, respectively, in persons with SCI (Bekhet et al. 2022; Dolbow et al. 2023a, 2023b). The current work is mechanistically aimed to unlock the black box regarding the role of electrical stimulation exercise on mitochondrial bioenergetics in persons with SCI. Based on our original work, we developed the hypothesis that changes in muscle size is likely to be associated with changes in mitochondrial density and activities to adjust for increasing metabolic demands (O'Brien et al. 2017c). The findings are likely to explore the effects of mitochondrial dysfunction in developing cardio-metabolic dysfunction in persons with SCI (O'Brien et al. 2017b). We previously noted that diminished mitochondrial density and activity are associated with increasing inflammatory cytokines and VAT CSA in persons with SCI (Goldsmith et al. 2022). Therefore, it is plausible to assume that decreasing VAT CSA following NMES-RT + FES would be associated with increasing mitochondrial density and activities in persons with SCI (Gorgey et al. 2023b).

The role of endurance exercise on mitochondrial proteins are well established for more than 5 decades now (Baldwin et al. 1972; Baar 2006). However, the role of resistance training is rather controversial with studies supporting the notion of dilution of mitochondrial density and activities with muscle hypertrophy whereas other studies refuted this notion (MacDougall et al. 1979; Ploutz et al. 1994; Pesta et al. 2011). Therefore, we provided two different paradigms of electrical stimulation exercise that represents both resistance and endurance type training for the paralyzed muscle in persons with SCI. NMES-RT preceded FES-LES to provide muscle conditioning by evoking skeletal muscle hypertrophy, decreasing intramuscular fat, increasing evoked torque and attenuating muscle fatigue (Gorgey et al. 2023b).

### Mitochondrial complex I after SCI

The findings of the current trial are highly empirical to our understanding of increasing prevalence of impaired glucose tolerance, insulin resistance and type II diabetes mellitus and development of cardiometabolic disorders in persons with SCI (Bauman and Spungen 1994; Lavela et al. 2006; Nash and Gater 2020). For the first time, we noted a defect in complex I enzyme activity in persons with SCI following training. Complex I activity did not change following NMES-RT + FES or PMT-FES at both time points.

The decrease in complex I activity is likely to contribute to excess NADH accumulation and redox imbalance of increasing NADH/NAD + (Victor et al. 2009; Hernandez-Mijares et al. 2011). This would eventually result in increasing oxidative stress and other metabolic disorders. Failure of utilization of NADH by complex I will result in low concentration of NAD + . The diminished level of NAD + would result in failure to induce deacetylation of specific target proteins (i.e. sirtuin proteins) that is necessary to attenuate development of type II DM (Antoun et al. 2015). Similar to previous findings (Chance et al. 1986; O'Brien et al. 2017b), it is safe to declare that complex I function is deficient in skeletal muscle of persons with SCI. Kelley et al. reported complex I is reduced in the muscle of type II DM (Kelley et al. 2002). Deficiency in complex I will result in excess accumulation of NADH and feedback inhibition of pyruvate dehydrogenase complex that will result in overproduction of reactive oxygen species. This dynamic process will result in complex I production of ROS and further protein degradations, mitochondrial dysfunction and cell death (Victor et al. 2009; Lefort et al. 2010; Hernandez-Mijares et al. 2011; Raza et al. 2011).

Another explanation is the CI assay is a stand method to measure complex I activity in isolated rat heart mitochondria (Chen et al. 2006), mouse heart mitochondria (Chen et al. 2024), and mouse brain mitochondria (Green et al. 2020). We believe that no/little CI activity of our SCI participants may be due to insufficient mitochondrial mass in their muscles. It is assumed that 25–50 ug of isolated mitochondria is needed to measure complex I activity (Green et al. 2020). In contrast, only need 2 ug isolated mitochondria is required to measure complex II and III activity. Thus, the activities of complex II and III can be easily detected in their muscles because it requires lower mitochondria compared to complex I assay.

### Adaptations of mitochondrial CS to exercise interventions

Citrate synthase (CS) is a biomarker of mitochondrial density and is a rate- limiting enzyme of the mitochondrial tricarboxylic acid (TCA) cycle. Most of the activities of CS occur inside the mitochondrial matrix. Deficiency in CS activity has been linked to several cardio-metabolic diseases within skeletal and heart muscles (Sumi et al. 2022). It is well established that endurance type training results in increasing mitochondrial density as measured by CS (Baldwin et al. 1972). On the contrary, resistance training has been shown to evoke muscle hypertrophy and increase in fiber CSA that would result in dilution of mitochondrial density (Ploutz et al. 1994). O’Brien et al. noted a strong relationship between skeletal muscle CSA and CS activities in persons with SCI (O'Brien et al. 2017b). Contrary, negative relationships were noted between CS and increasing VAT or intramuscular fat CSAs (O'Brien et al. 2017a). It is obvious that CS is likely to be related to markers of carbohydrate and lipid metabolisms in persons with SCI (O'Brien et al. 2017a, b). Previously, we noted that 16 weeks of NMES-RT and TT resulted in 29% increase in fiber CSA with subsequent increase in CS and succinate dehydrogenase (Gorgey et al. 2020). In the current trial, we noted that evoking muscle hypertrophy following 12 weeks either NMES-RT or FES-LEC would result in subsequent increase in CS. Surprisingly, there was no increase in CS following the addition of 12 weeks of FES-LEC to 12 weeks of NMES-RT. This is concurrent with our recent findings that indicated that such addition of FES-LEC to NMES-RT did not evoke further muscle hypertrophy in persons with SCI (Gorgey et al. 2023b).

To highlight the role of muscle hypertrophy on CS, we calculated the individual differences in CS at P1 and P2 compared to BL (Fig. 2). This would allow each participant to serve as his own control. The entire paradigm of CS has changed from P1 to P2 once FES-LEC was added to PMT in the PMT + FES group; highlighting the significance of evoking muscle hypertrophy in persons with SCI on mitochondrial density. This may provide additional insights on the paracrine role of the paralyzed skeletal muscle following conditioning with either NMES-RT or FES-LES. Muscle hypertrophy is likely to serve as a countermeasure of insulin resistance and dyslipidemia via increasing mitochondrial density. The observation is supported by pre-established relationships between CS and either carbohydrate or lipid metabolism in persons with SCI (O'Brien et al. 2017b).

### Adaptations of mitochondrial complexes to exercise interventions

The evidence of RT on mitochondrial biogenesis is still controversial (Groennebaek and Vissing 2017). Contrary to the findings of the current trial, Porter et al. noted that 12 weeks, 3 × weekly, of RT resulted in 11% increase in complex I protein activities without changes in other complexes (II-V). In the current trial, we adopted an exercise frequency of 2 × weekly which previously demonstrated robust muscle hypertrophy in persons with SCI (Ryan et al. 2013; Gorgey et al. 2021). However, others indicated a training frequency of 5 times weekly are necessary to increase the ETC enzymatic activities compared to 3 × weekly in a murine model (Chilibeck et al. 1998). In agreement, others noted that a duration of 16–24 weeks of exercise training are likely to increase the ETC activities of complex I and IV (Menshikova et al. 2005; Boveris and Navarro 2008). The increase in the activities of ETC is likely explained by enhanced downstream expression of PGC-1 alpha which subsequently enhanced mitochondrial biogenesis to meet the increase in cellular demands (Baar et al. 2002). We have previously noted overexpression of PGC-1 alpha following 16 weeks of NMES-RT and TT (Gorgey et al. 2020, 2023a); which may explain the superior effects of NMES-RT + FES group compared to PMT + FES on mitochondrial biogenesis. RT resulted in recognized ability to enhance mitochondrial biogenesis especially in older adults and persons with chronic medical disorders (Groennebaek and Vissing 2017). Furthermore, low-load RT exercise, similar to the current NMES-RT paradigm, is accompanied with greater mitochondrial biogenesis compared to high load RT (Groennebaek & Vissing 2017).

### Strengths and limitations

The current trial enrolled 32 participants with chronic SCI. Persons underwent three muscle biopsies with 12 weeks apart to examine the effects of two different paradigms of electrical stimulation exercise on mitochondrial bioenergetics. We considered our trial to be one of the largest randomized clinical trials that addressed the effects of lab-based training on mitochondrial health after SCI. Unlike other trials, we did not measure mitochondrial respiration because of limited resources. We have previously measured mitochondrial respiration of isolated or permeabilized muscle fibers (Lai et al. 2022). However, mitochondrial respiration required fresh muscle fibers and not frozen samples compared to what has been used in the current trial. Mitochondrial respiration is laborious and require a highly dedicated research team to undergo muscle biopsy as well as to run mitochondrial respiration on the same day (Lai et al. 2022). However, a recent study demonstrated the ability to use frozen muscle tissue homogenates to measure mitochondrial respiration of complexes I and II in the murine model (Ebanks et al. 2023).

Frozen sample biopsies provided a unique experience to study protein and RNA expressions following muscle hypertrophy in persons with SCI (Gorgey et al. 2023a). Compared to our earlier work, we did expand our measures to include complexes I, II, and III as well as NFR and NCR. However, we did not measure complex IV because of limited muscle biopsy size that were available to run complex IV. We have previously measured complex IV in the permeabilized muscle biopsy fibers (Lai et al. 2022).

In general, the current results should be considered with caution. SCI population is a heterogeneous in nature as result of wide age range, level of injury and severity of injury as demonstrated in the current study. Furthermore, the inclusion of females may have resulted in difficulty interpreting the results; currently the effects of sex on mitochondrial enzymatic activities is not well established after SCI (Miotto et al. 2018). However, the sex distribution agreed with the National Statistical Center and yielded similar distribution of 4 males:1 female (National Spinal Cord Injury Statistical Center 2023). We have also adopted MANCOVA as the primary statistical approach to ensure that we accounted for baseline variabilities between both groups.

## Summary/conclusion

This is the first study to comprehensively examine the effects of combined RT and endurance type training on mitochondrial density and mitochondrial ETC activities after SCI. Our results indicated a very low complex I activity which is the entry gate for the oxidation of substrates by ETC. Additionally, our training paradigms suggested that evoking muscle hypertrophy via either NMES-RT or FES-LEC may potentially enhance mitochondrial density and mitochondrial ETC activities (Complexes II and III). The addition of FES-LEC following NMES-RT maintained the gains in CS and complexes II and III; whereas clearly showed increase in these mitochondrial parameters following 12 weeks of PMT. NMES-RT may have superior effects on mitochondrial ETC activities without additional increases following FES-LEC; especially pertaining to complex III activity. This may suggest safe home applications without the need of reliance on expensive ergometers to enhance mitochondrial bioenergetics in persons with SCI.

## Data Availability

The data will not be publicly available and only data will be available upon written request to the corresponding authors.

## Abbreviations

ASIA: American Spinal Injury Association (ASIA)
AIS: ASIA Impairment Scale
BL: Baseline
BMI: Body mass index
CSA: Cross-sectional area
Complex I: NADH:ubiquinone oxidoreductase
Complex II: Succinate:ubiquinone oxidoreductase
Complex III: Ubiquinol-cytochrome c oxidoreductase
FES: Functional electrical stimulation
FES-LES: Functional electrical stimulation-lower extremity cycling
ISNCSCI: International Standards for Neurological Classification of Spinal Cord Injury
IMF: Intramuscular fat
MRI: Magnetic resonance imaging
NCR: NADH cytochrome c oxidoreductase
NFR: NADH-ferricyanide oxido-reductase
NMES: Neuromuscular electrical stimulation
NMES-RT: Neuromuscular electrical stimulation-resistance training
PMT: Passive movement training
P1: Post-intervention 1
P2: Post-intervention 2
RPM: Revolution per minute
SCI: Spinal cord injury
TT: Testosterone treatment
